# Relationship between skeletal bone mineral density and subjective masticatory difficulty

**DOI:** 10.1186/s12903-022-02172-8

**Published:** 2022-04-21

**Authors:** Seok Woo Hong, Jeong-Hyun Kang

**Affiliations:** 1 Department of Orthopedic Surgery, Kangbuk Samsung Hospital, Sungkyunkwan University School of Medicine, 29, Saemunan-ro, Jongno-gu, Seoul, 03181 Republic of Korea; 2 Clinic of Oral Medicine and Orofacial Pain, Institute of Oral Health Science, Ajou University School of Medicine, 164, Worldcup-ro, Yeongtong-gu, Suwon, Gyeonggi-do 16499 Republic of Korea

**Keywords:** Bone mineral density, Elderly, Mastication, Osteoporosis

## Abstract

**Background:**

Masticatory ability is an essential factor for sustaining quality of life and social and systemic well-being, particularly in elderly. This study aimed to reveal the association between subjective masticatory difficulty and skeletal bone mineral density (BMD).

**Methods:**

Data from the Korean National Health and Nutrition Examination Survey, which was conducted from 2008 to 2011 were analyzed. This study included 13,092 Koreans (5656 males, 7436 females) over 50 years of age. Masticatory difficulty was evaluated based on a self-reported questionnaire. Areal BMD of the total hip, femoral neck, and lumbar spine as well as lean body mass were determined using dual-energy X-ray absorptiometry. Data about the sociodemographic characteristics, physical activity, number of teeth present, sum of decayed, missing, and filled permanent teeth (DMFT) index and Community Periodontal Index (CPI) were collected. Multivariate logistic regression analysis was conducted to analyze associations between subjective masticatory difficulty and BMD, adjusting for the confounding covariates.

**Results:**

Significant differences were observed in the areal BMD of the total hip, femoral neck, and lumbar spine as well as lean body mass accordance with the presence of subjective masticatory difficulty in both males and females. The number of teeth, DMFT, and CPI score did not show significant differences based on the presence of self-reported satisfaction of chewing performance in both males and females. Results from multivariate logistic regression demonstrated that the subjective masticatory difficulty showed significant interactions with skeletal BMD and the associations between masticatory satisfaction and BMD of the total hip and femoral neck were more prominent in females compared to those in males.

**Conclusions:**

The skeletal BMD, particularly areal BMD of the femoral neck was significantly associated with subjective masticatory difficulty in elderly, especially in elder females.

## Background

Masticatory ability is not only a determinant of oral health but also a reflection of the quality of life and social and systemic well-being, particularly in elderly [1, 2]. Chewing difficulty in the elderly causes nutritional deficiencies and gastrointestinal disturbances which are associated with greater risk of frailty and mortality [3–8]. Recent studies have focused on the association among masticatory performance, systemic frailty, and diverse conditions related to the aging such as decreased resilience, cognitive impairment, depression, cardiovascular disease, and sarcopenia [9–13].

Chewing performance is a complex process which involves intact dentition, integrated oral mucosa, sufficient salivary secretion, healthy temporomandibular joints, and coordination of sensory stimuli, motor nerve activation, and proper response of muscle and other connective tissue structures [14–18]. Rehabilitation of masticatory function is one of the main goals of dental treatment, and several previous works have attempted to reveal not only the associated local factors such as tooth loss, periodontitis, decreased masticatory force, temporomandibular disorders, dry mouth, and abnormal occlusal relationships but also related systemic factors including sarcopenia and osteoporosis [9, 19].

Spontaneous bone loss is one of the main features of aging and can increase the risk of osteoporotic fracture and mortality [20, 21]. Several studies have investigated the link between oral health, status of dentition, and skeletal bone mineral density (BMD) [19, 22–27]. Osteoporotic bone changes may affect alveolar bone density and ridge resorption rate and this, in turn, could lead to tooth loss and impaired masticatory function [23, 28–32]. Moreover, poor oral condition could result in improper nutritional intake that could also lead to osteoporosis [33]. The fragmentary knowledge of the relationship between number of teeth, alveolar bone density, periodontal health condition, and skeletal BMD has been reported several times previously. However, to the best of our knowledge, a compressive and integrated understanding of the interaction of masticatory performance and osteoporosis has not elucidated, so far. Therefore, we hypothesis that masticatory dysfunction would have critical role in determining skeletal BMD in elderly.

Even though, several previous studies have attempted to determine the relationships between skeletal BMD and masticatory function, the relatively small sample sizes of those studies inevitably have limited the validity of the results. The Korea National Health and Nutrition Examination Survey (KNHANES) conducted by the Korean center for Disease Control and Prevention which annually monitors the general health, oral condition, and nutritional status of the South Korean population includes a relatively large number of samples, therefore can provide valid and meaningful results. Hence, the aim of the present study was to investigate the relationships between self-reported masticatory difficulty and skeletal BMD after considering the possible confounders in a nationally representative sample of the Korean population.

## Materials and methods

### Study population

The present study was based on the data obtained from the 2008 to 2011 KNHANES, a nationally representative survey conducted by the Korean Center for Disease Control and Prevention. This study enrolled 13,092 Korean subjects (5656 males, 7436 females; mean age 64.1 ± 0.1; age range 50–80 years) over 50 years of age. To enroll a representative sample from the population, a stratified, multistage, and clustered probability approach was used. The survey consisted of a nutritional survey, individual interview, and health examination survey. Data were obtained via household interviews from direct standardized physical examinations. Trained interviewers performed the interviews using structured questionnaires. Written informed consents were provided from all participants. Only data from responders aged over 50-year-old were included.

### Chewing discomfort and oral-health associated parameters

A trained interviewer interviewed the participants using a structured questionnaire. Subjective masticatory discomfort was assessed on a 5 points scale (very uncomfortable, uncomfortable, fair, comfortable, and very comfortable). For the analysis, masticatory difficulties were classified as follows: no problem (very comfortable, comfortable, fair) and problem (uncomfortable, very uncomfortable).

The oral examination was conducted by trained dentists. Periodontal health status was examined using the Community Periodontal Index (CPI) based on the criteria given by the World Health Organization [34]. The CPI score was as follows: 0, healthy gingiva; 1, gingival bleeding; 2, presence of calculus; 3, pocket depth of 3.5–5.5 mm; 4, pocket depth of 5.5 mm or more. The ten index teeth were #11, 16, 17, 26, 27, 31, 36, 37, 46, and 47. If no index tooth was present in a sextant qualifying for examination, the adjacent remaining tooth in that sextant was selected. The number of teeth present and the sum of decayed, missing, and filled permanent teeth (DMFT) index were also determined [35].

### Anthropometric measurement

All participants put on uniformed light gown which provided by the survey staff without shoes and trained examiners assessed their weight (kg) and height (cm). The body mass index (BMI) was evaluated by dividing the weight (kg) by the square of the height (m) [36].

### BMD measurement

Whole body dual-energy X-ray absorptiometry (DEXA) was performed with a QDR Discovery fan beam densitometer (Hologic, Bedford, MA, USA), as per the procedures recommended by the manufacturer. The areal BMDs (aBMDs) of the total hip, femoral neck, and lumbar spine were measured with DEXA. The aBMD was calculated as the amount of bone mineral contents divided by the bone scanned area [37]. The DEXA results were analyzed using the standard techniques of the Korean Society of Osteoporosis and Hologic Discovery software (version 13.1; Hologic Inc., Bedford, MA, USA).

### Sociodemographic factors and health-related behaviors

The data about sociodemographic factors and health-related behaviors including smoking, alcohol drinking, menopause and physical activities were assessed by self-administered questionnaires from KNHANES. Monthly household income was adjusted for the number of household members and classified into following four different quartiles. Education level was also classified into four groups based on the Korean education system: elementary school (less than 6 years of institutionalized education), middle school (7–9 years of institutionalized education), high school (10–13 years of institutionalized education), and over college education (more than 14 years of education).

Cigarette smoking was categorized into following three groups: nonsmoker, smokers who have smoked at least 5 packs in their entire lives, and smokers who currently smoke and have smoked more than 5 packs in their entire lives. Alcohol drinking was classified into following two groups; none or light drinker (0–3 day/month) and moderate to heavy drinker (> 4 days/month).

In females, the information about whether menopause had occurred were collected. Physical activity level was measured using the Korean version of the International Physical Activity Questionnaire (IPAQ) short form [38]. The respondents were classified as performing high/moderate intensity physical activity longer than 30 min and more than 5 times/week, respectively [38].

### Metabolic syndrome

Metabolic syndrome was diagnosed if participants had at least three fulfilling criteria among the following five criteria as previous reports suggested [39, 40]: (1) a clinical diagnosis of diabetes treated with insulin/oral hypoglycemic medication or a fasting serum glucose level of more than 110 ml/dL; (2) current use of antihypertensive medication or arterial blood pressure of more than 130/85 mm Hg; (3) plasma triglyceride level of more than 150 mg/dL; (4) high-density lipoprotein cholesterol level of less than 40 mg/dL for males or less than 50 mg/dL for females; or (5) a waist size greater than 90 cm for males or 80 cm for females.

### Statistical analysis

All statistical analysis was conducted using a complex design including stratification, clustering and weighting. Sample weights were constructed for sample participants to represent the Korean population by accounting for the complex survey design, survey nonresponse, and stratification (according to geographic area, age, and sex). All values were considered significant when *P* < 0.05.

All analysis was conducted separately for male and females owing to the different rate of background bone metabolism and muscle mass between the sexes. Subjective masticatory difficulty was an outcome variable and aBMDs of the total hip, femoral neck, and lumbar spine were the main explanatory variables. Rao-Scott chi-square and independent t test were used to compare the differences in the demographic factors, skeletal BMD, number of teeth, DMFT, CPI, and information about menopause and physical activity for categorical and continuous variables, respectively accordance with the presence of subjective masticatory difficulty. Multivariate logistic regression analysis was used to analyze the interactions between self-reported masticatory difficulty and skeletal aBMD adjusted for the potential confounders. Owing to high collinearity among aBMD of the total hip, femoral neck, and lumbar spine, separate analysis of aBMD of the total hip, femoral neck, and lumbar spine was conducted. Model 1 was unadjusted, while Model 2 was adjusted for age and BMI. Model 3 was adjusted for age, BMI, and general health status and behaviors including smoking, drinking, and presence of metabolic syndrome. Model 4 was adjusted for age, BMI, general health status and behaviors, physical activity, experience of menopause for females, and socioeconomic factors, including smoking, drinking, presence of metabolic syndrome, and levels of household income and education.

## Results

The differences in sociodemographic factors including age, household income, educational level, and smoking status were statistically significant between participants with subjective masticatory difficulty and those without it in both males and females. On the other hand, presence of metabolic syndrome and alcohol consumption in males and BMI in females did not show significant differences. No significant differences of performing high/moderate intensity physical activity were detected in two groups in both males and females. The number of female participants with menopause was significantly higher in females without subjective masticatory difficulty compared to those with it despite of higher BMD in females without masticatory dissatisfaction (Table 1).

**Table 1:** The demographic characteristics of the subjects according to masticatory difficulty

		Masticatory difficulty				
Variable	Total N	No		Yes		*P* value
		N	% (95% CI)	N	% (95% CI)	
Male						
Age† (years) (Mean ± SE)	5531	3062 (55.7%)	63.0 ± 0.2	2469 (44.3%)	64.4 ± 0.2	< 0.001**
BMI† (kg/m^2^) (Mean ± SE)	5502	3048 (56.4%)	23.9 ± 0.7	2454 (43.6%)	23.3 ± 0.7	< 0.001**
Household income	5320					< 0.001**
< 25%		744	24.7 (22.8–26.6)	855	35.3 (32.8–37.9)	
25–49%		773	26.5 (24.6–28.5)	621	27.0 (24.8–29.3)	
50–74%		651	21.9 (20.2–23.7)	474	19.6 (17.7–21.6)	
≥ 75%		778	26.9 (24.8–29.2)	424	18.1 (16.2–20.2)	
Education	5656					< 0.001**
≤ primary school		914	31.0 (28.8–33.3)	1041	42.9 (40.3–45.5)	
Middle school		586	19.6 (17.9–21.4)	500	20.4 (18.6–22.3)	
High school		838	28.6 (26.6–30.7)	557	24.2 (22.1–26.3)	
≥ College or higher		929	20.8 (18.8–23.1)	291	12.6 (10.8–14.5)	
Metabolic syndrome	2549					0.265
Yes		270	18.0 (15.7–20.4)	196	16.1 (13.8–18.7)	
No		1162	82.0 (79.6–84.3)	921	83.9 (81.3–86.2)	
Smoking status	5368					< 0.001**
Never of former		63	2.0 (1.5–2.7)	177	7.5 (6.3–8.9)	
≤ 5 packs		2353	78.6 (76.7–80.3)	2215	92.4 (91.0–93.5)	
≥ 5 packs		554	19.1 (17.4–20.9)	3	0.1 (0–0.4)	
Alcohol consumption	5366					0.183
None or light		256	9.1 (8.0–10.4)	177	7.5 (6.3–8.9)	
Moderate or heavy		2718	90.7 (89.4–91.9)	2215	92.4 (91.0–93.5)	
High intensity physical activity	5471					0.131
Yes		522	18.7 (16.9–20.5)	378	16.6 (14.7–18.7)	
No		2510	81.3 (79.5–83.1)	2061	83.4 (81.3–85.9)	
Moderate intensity physical activity	5471					0.802
Yes		392	12.4 (10.9–14.1)	307	12.1 (10.7–13.8)	
No		2641	87.6 (85.9–89.1)	2131	87.9 (86.2–89.3)	
Female						
Age† (years) (Mean ± SE)	7436	4051 (54.4%)	62.5 ± 0.2	3385 (45.6%)	66.1 ± 0.2	< 0.001**
BMI† (kg/m^2^) (Mean ± SE)	7405	4037 (54.4%)	24.2 ± 0.6	3368 (45.6%)	24.2 ± 0.1	0.556
Household income	7084					< 0.001**
< 25%		1193	29.9 (27.9–32.0)	1457	45.7 (43.2–48.3)	
25–49%		988	25.5 (23.7–27.3)	816	24.8 (22.9–26.8)	
50–74%		798	20.3 (18.7–21.9)	526	16.5 (14.8–18.3)	
≥ 75%		899	24.4 (22.2–26.7)	407	13.0 (11.4–14.8)	
Education	7143					< 0.001**
≤ primary school		2236	56.8 (54.4–59.3)	2463	76.4 (74.2–78.5)	
Middle school		629	16.2 (14.8–17.7)	379	11.9 (10.5–13.5)	
High school		772	20.1 (18.4–21.9)	322	9.4 (8.1–10.9)	
≥ College or higher		264	6.9 (5.6–8.5)	78	2.2 (1.7–3.0)	
Menopause	5337					< 0.001**
Yes		1090	41.4 (39.1–43.8)	661	30.2 (27.8–32.7)	
No		1712	58.6 (56.2–60.9)	1874	69.8 (67.3–72.2)	
Metabolic syndrome	3365					0.029*
Yes		319	15.9 (14.0–18.1)	289	19.6 (17.1–22.3)	
No		1549	84.1 (81.9–86.0)	1208	80.4 (77.7–82.9)	
Smoking status	7150					< 0.001**
Never of former		3625	92.6 (91.3–93.5)	2900	88.8 (87.0–91.1)	
≤ 5 packs		29	0.8 (0.4–1.0)	35	1.3 (0.7–1.8)	
≥ 5 packs		243	6.6 (54.4–7.6)	318	9.9 (8.5–11.3)	
Alcohol consumption	7157					< 0.001**
None or light		1295	33.1 (31.3–35.0)	1243	39.6 (37.2–42.0)	
Moderate or heavy		2609	66.6 (64.7–68.5)	2010	60.0 (57.6–62.4)	
High intensity physical activity						0.486
Yes	7347	446	11.6 (10.4–13.0)	378	11.0 (9.7–12.5)	
No		3556	88.4 (87.0–89.6)	2967	89.0 (87.5–90.3)	
Moderate intensity physical activity						0.853
Yes	7347	503	12.4 (11.2–13.8)	479	12.6 (11.2–14.1)	
No		3500	87.6 (86.2–88.8)	2865	87.4 (85.9–88.8)	

CI, confidential interval; SE, standard error

%: Weighted percentage by column

Data obtained from Rao-Scott chi-square test

^†^Data obtained from independent T-test and descriptive values are shown as mean ± SE

^*^ P < 0.05, ** *P* < 0.001 by Rao-Scott Chi-Square test or independent T-test

The significant differences of aBMD and T score of the total hip, femoral neck, and lumbar spine in accordance with presence of subjective masticatory difficulty was detected in both males and females. The number of teeth, DMFT, and CPI score did not show significant differences in accordance with the existence of subjective masticatory difficulty in both males and females (Table 2).

**Table 2:** Bone mineral density, number of teeth, DMFT, periodontal status, and hormonal levels according to masticatory difficulty

		Masticatory difficulty				*P* value
Variable	Total N	No		Yes		
		N	Mean ± SE or % (95% CI)	N	Mean ± SE or % (95% CI)	
Male						
Number of teeth	3242	1907 (58.2%)	12.8 ± 0.4	1355 (41.8%)	12.3 ± 0.5	0.382
DMFT	3234	1886 (58.2%)	5.93 ± 0.17	1348 (41.8%)	5.88 ± 0.21	0.858
CPI	2340					0.516
0		304	23.7 (19.2–28.9)	238	24.9 (19.5–31.1)	
1		60	4.9 (3.0–7.8)	41	5.5 (3.2–9.4)	
2		415	29.0 (24.5–34.0)	242	23.9 (19.4–29.1)	
3		477	35.6 (30.7–40.8)	370	38.2 (32.5–44.3)	
4		118	6.9 (4.6–10.1)	75	7.4 (3.7–14.5)	
Total hip aBMD (g/cm^2^)	2762	1570 (58.2%)	0.930 ± 0.004	1192 (41.8%)	0.910 ± 0.005	< 0.001**
Total hip T score	2762	1570 (58.2%)	− 0.090 ± 0.028	1192 (41.8%)	− 0.220 ± 0.035	< 0.001**
Femoral neck aBMD (g/cm^2^)	2762	1570 (58.2%)	0.750 ± 0.004	1192 (41.8%)	0.730 ± 0.004	< 0.001**
Femoral neck T score	2762	1570 (58.2%)	− 0.800 ± 0.030	1192 (41.8%)	− 0.940 ± 0.035	< 0.001**
Lumbar spine aBMD (g/cm^2^)	2690	1537 (58.3%)	0.940 ± 0.004	1153 (41/7%)	0.930 ± 0.006	0.040*
Lumbar spine T score	2690	1537 (58.3%)	− 0.680 ± 0.037	1153 (41.7%)	− 0.790 ± 0.049	0.040*
Lean body mass (kg)	2698	1536 (58.2%)	50.6 ± 0.2	1162 (41.8%)	49.4 ± 0.3	< 0.001**
Female						
Number of teeth	4373	2596 (60.0%)	12.5 ± 0.4	1777 (40.0%)	12.1 ± 0.5	0.449
DMFT	4351	2570 (59.7%)	5.82 ± 0.16	1781 (40.3%)	6.00 ± 0.21	0.436
CPI^†^	3043					0.053
0		408	21.7 (17.4–26.6)	333	24.3 (18.9–30.7)	
1		75	3.7 (2.4–5.7)	78	6.2 (3.9–9.5)	
2		548	30.2 (25.5–35.4)	286	24.7 (20.0–30.1)	
3		630	34.6 (29.4–40.2)	443	35.9 (30.2–42.0)	
4		151	9.8 (6.4–14.9)	91	8.9 (5.5–14.1)	
Total hip aBMD (g/cm^2^)	3749	2126 (55.7%)	0.800 ± 0.003	1623 (44.3%)	0.750 ± 0.004	< 0.001**
Total hip T score	3749	2126 (55.7%)	− 0.470 ± 0.029	1623 (44.3%)	− 0.840 ± 0.039	< 0.001**
Femoral neck aBMD (g/cm^2^)	3749	2126 (55.7%)	0.640 ± 0.003	1623 (44.3%)	0.600 ± 0.004	< 0.001**
Femoral neck T score	3749	2126	− 1.48 ± 0.03	1623	− 1.89 ± 0.04	< 0.001**
Lumbar spine aBMD (g/cm^2^)	3645	2075 (56.0%)	0.830 ± 0.004	1570 (44.0%)	0.780 ± 0.005	< 0.001**
Lumbar spine T score	3645	2075 (56.0%)	− 1.56 ± 0.04	1570 (44.0%)	− 1.93 ± 0.04	< 0.001**
Lean body mass (kg)	3679	2087 (55.7%)	37.0 ± 0.1	1592 (44.3%)	36.3 ± 0.2	< 0.001**

ALP, alkaline phosphatase; aBMD, areal bone mineral density; CI, confidential interval; CPI, community periodontal index; DMFT, decay, missing, filling tooth; PTH, parathyroid hormone; SE, standard error

%: Weighted percentage by column

Data obtained from independent T-test

Descriptive values are shown as mean ± SE

^†^Data obtained from Rao-Scott Chi-square test and descriptive values are show as % (95% CI)

^*^ P < 0.05, ** *P* < 0.001 by independent T-test and Rao-Scott Chi-square test

Results from multivariate logistic regression demonstrated that the interactions between skeletal BMD and subjective masticatory difficulty were more prominent in females compared to those in males. Significant associations between subjective masticatory difficulty and aBMD of the total hip, femoral neck, and lumbar spine were detected in only Model 1 in males. Otherwise, significant interactions with aBMD of the femoral neck and self-reported masticatory difficulty were observed in Model 1, 2, 3, and 4 in females. aBMD of the total hip also showed significant associations with subjective masticatory difficulty in Model 1, 2, and 3 and aBMD of the lumbar spine showed significant relationships in Model 1 and 2 in females (Table 3).

**Table 3:** Adjusted association between masticatory difficulty and bone mineral density

	Model 1	Model 2	Model 3	Model 4
*Male*				
Total hip aBMD				
Odd ratio (95% CI)	2.781 (1.511–5.117)	1.177 (0.548–2.528)	1.186 (0.374–3.762)	0.419 (0.170–1.030)
B ± SE	1.023 ± 0.310	0.163 ± 0.389	0.171 ± 0.587	− 0.871 ± 0.458
*P* value	< 0.001**	0.675	0.771	0.058
R square (Nagelkereke)	0.006	0.016	0.058	0.164
Femoral neck aBMD				
Odd ratio (95% CI)	3.259 (1.711–6.205)	1.410 (0.651–3.054)	1.024 (0.356–4.072)	0.554 (0.223–1.376)
B ± SE	1.181 ± 0.327	0.344 ± 0.393	0.186 ± 0.619	− 0.591 ± 0.463
* P* value	< 0.001**	0.382	0.764	0.202
R square (Nagelkereke)	0.007	0.016	0.058	0.163
Lumbar spine aBMD				
Odd ratio (95% CI)	1.727 (1.022–2.918)	1.189 (0.666–2.122)	1.096 (0.474–2.535)	0.651 (0.301–1.409)
B ± SE	0.546 ± 0.267	0.173 ± 0.294	0.092 ± 0.426	− 0.430 ± 0.393
* P* value	0.041*	0.557	0.829	0.275
R square (Nagelkereke)	0.002	0.013	0.057	0.159
*Female*				
Total hip aBMD				
Odd ratio (95% CI)	15.9 (8.1–31.4)	2.729 (1.083–6.877)	4.848 (1.084–21.686)	3.788 (0.810–17.717)
B ± SE	2.767 ± 0.345	1.004 ± 0.470	1.579 ± 0.762	1.332 ± 0.784
P value	< 0.001**	0.033*	0.039*	0.090
R square (Nagelkereke)	0.038	0.069	0.085	0.134
Femoral neck aBMD				
Odd ratio (95% CI)	29.615(13.964–62.806)	4.808 (1.757–13.161)	9.477 (1.883–47.698)	6.644 (1.207–36.560)
B ± SE	3.388 ± 0.382	1.570 ± 0.512	2.249 ± 0.822	1.727 ± 0.852
* P* value	< 0.001**	0.002*	0.007*	0.030*
R square (Nagelkereke)	0.047	0.071	0.089	0.137
Lumbar spine aBMD				
Odd ratio (95% CI)	7.313 (4.129–12.953)	2.462 (1.270–4.774)	1.923 (0.784–4.716)	0.999 (0.374–2.666)
B ± SE	1.990 ± 0.291	0.901 ± 0.337	0.654 ± 0.456	− 0.001 ± 0.027
* P* value	< 0.001**	0.008*	0.153	0.998
R square (Nagelkereke)	0.027	0.063	0.075	0.125

Model 1: Unadjusted

Model 2: Adjusted for age and BMI

Model 3: Adjusted for age, BMI, and general health status and behaviors including smoking, drinking, and metabolic syndrome

Model 4: Adjusted for age, BMI, general health status and behaviors, physical activity, experience of menopause for females, and socioeconomic factors, including smoking, drinking, and metabolic syndrome, levels of household income and education

^*^ P < 0.05, ** *P* < 0.001 by multivariate logistic regression

## Discussion

The aim of this study was to reveal the associations between masticatory difficulty and skeletal BMD in elder populations using the KNHANES data in 2008–2011 with large number of samples from an authorized institution after adjusting for confounding covariates. The main finding from the present study was significant association between skeletal BMD and subjective masticatory function in the elder populations, particularly in females. Oral hypofunction in elderly could have impact on maintaining bone and muscle mass through diverse pathways including insufficient nutritional intake and inflammatory mechanisms [41–43]. The mechanical forces applied to the bone that originate from associated muscles are crucial to maintain skeletal health and bony integrity [44–46]. Therefore, oral hypofunction including masticatory dysfunction could have influence on decreased muscle and bone mass and deteriorated muscle mass and function also have impacts on maintaining bone mineral density through bone-muscle interactions.

Previous studies have shown conflicting results about the sex differences in the interactions between skeletal BMD and masticatory efficiency. One study demonstrated more prominent influences of osteoporosis on lower masticatory efficiency in females compared to those in males [19], otherwise another study showed significant relationships between masticatory dysfunction and osteoporosis, particularly in male elderly [24]. Both animal and human studies revealed that osteoporosis may affect alveolar bone loss and tooth loss, especially in post-menopausal females with increased bone turnover rates [28–32]. The mechanical disadvantages of subchondral bone in the mandibular condyles of females, including more fragile characteristics for static and dynamic loading compared to those in males also have been revealed [47]. Masticatory ability in post-menopausal females with relatively higher bone turnover rates, more fragile subchondral local bone structure, and lower skeletal and masticatory muscle mass compared to males [48] might be more sensitively influenced by the changes in the skeletal BMD. Hence, inadequate nutritional intake owing to masticatory dysfunction would lead to accelerated bone loss and osteoporosis and this changes would be more prominent with post-menopausal females with increased bone turnover rates.

The aforementioned results exhibited that there were strong significant relationships between aBMD of the femoral neck and total hip and subjective masticatory difficulties. The aBMD of the lumbar spine would be influenced by several factors including posture and arthritic changes of the spine [49–51], so adopting aBMD of the total hip or femoral neck would be recommended for proper evaluation of skeletal BMD and diagnosis of osteoporosis. The lesser significance between aBMD of the lumbar spine and subjective masticatory difficulties could be owing to those factors.

The results from this study demonstrated a lack of significant differences in the number of remaining teeth, DMFT, and CPI score, accordance with the presence of subjective masticatory difficulty. Several previous studies reported the role of number of remaining and functional teeth and periodontal health on the maintenance of chewing ability, particularly in the elderly [52–57]. Chewing function can be evaluated by objective clinical tests or self-reported measures [58]. Generally, objective chewing efficiency which was different concept from subjective masticatory satisfaction seemed to be critically influenced by the number of remaining and functional teeth, status of prosthodontics, and conditions of periodontal health but subjective masticatory satisfaction showed conflicting results [9, 52, 59]. However, to the best of our knowledge, no universally adopted golden standard for determination of masticatory difficulty have been proposed. The perception of masticatory satisfaction could be affected by other factors besides oral health status, such as depression, resilience, ability of physical performances, and coordination of tongue activity [9, 59, 60] but subjective masticatory difficulty still has its own significant value in geriatric medicine and dentistry. Subjective chewing satisfaction plays an important role in elderly because not only objective chewing efficiency but also subjective masticatory satisfaction could have a role in the prediction of progression of frailty in elder population [7].

The present study has several limitations. First of all, owing to the retrospective cross-sectional study design, the causal relationships between osteoporosis and masticatory difficulties could not be derived. Secondly, lack of information about other oral health associated factors related to the chewing function, including salivary flow rate, occlusal relationships, masticatory force, and temporomandibular disorders by proper diagnostic criteria inevitably compromise the significance of the results from the study. Thirdly, due to the retrospective study design, precise information about participants, such as underlying diseases and medication history could not be provided.

## Conclusions

In conclusion, the skeletal BMD, particularly aBMD of the femoral neck was significantly associated with subjective masticatory difficulty in elderly, especially in elder females. The importance of maintaining oral health in elder patients would be emphasized to prevent osteoporosis and this might lead to prevention of frailty in long terms. Understanding this interaction would be warranted for dentist and physicians for better management of frailty and for successful aging.

## Data Availability

The datasets used and/or analyzed during the current study are available from the homepage of Korean Center for Disease Control and Prevention (http://knhanes.kdca.go.kr) with no restriction apply to the availability of these data.

## Abbreviations

BMD: Bone mineral density (BMD)
CPI: Community Periodontal Index
DEXA: Dual-energy X-ray absorptiometry
DMFT: Decayed, missing, and filled permanent teeth
KNHANES: The Korea National Health and Nutrition Examination Survey
